# Characterization of the structural forces governing the reversibility of the thermal unfolding of the human acidic fibroblast growth factor

**DOI:** 10.1038/s41598-021-95050-2

**Published:** 2021-08-02

**Authors:** Shilpi Agrawal, Vivek Govind Kumar, Ravi Kumar Gundampati, Mahmoud Moradi, Thallapuranam Krishnaswamy Suresh Kumar

**Affiliations:** Department of Chemistry and Biochemistry, University of Arkansas, Fayetteville, AR USA

**Keywords:** Biochemistry, Computational biology and bioinformatics

## Abstract

Human acidic fibroblast growth factor (hFGF1) is an all beta-sheet protein that is involved in the regulation of key cellular processes including cell proliferation and wound healing. hFGF1 is known to aggregate when subjected to thermal unfolding. In this study, we investigate the equilibrium unfolding of hFGF1 using a wide array of biophysical and biochemical techniques. Systematic analyses of the thermal and chemical denaturation data on hFGF1 variants (Q54P, K126N, R136E, K126N/R136E, Q54P/K126N, Q54P/R136E, and Q54P/K126N/R136E) indicate that nullification of charges in the heparin-binding pocket can significantly increase the stability of wtFGF1. Triple variant (Q54P/K126N/R136E) was found to be the most stable of all the hFGF1 variants studied. With the exception of triple variant, thermal unfolding of wtFGF1 and the other variants is irreversible. Thermally unfolded triple variant refolds completely to its biologically native conformation. Microsecond-level molecular dynamic simulations reveal that a network of hydrogen bonds and salt bridges linked to Q54P, K126N, and R136E mutations, are responsible for the high stability and reversibility of thermal unfolding of the triple variant. In our opinion, the findings of the study provide valuable clues for the rational design of a stable hFGF1 variant that exhibits potent wound healing properties.

## Introduction

Protein folding has been a fascinating puzzle that has attracted the attention of researchers for a long time^1^. However, the mechanism by which a nascent polypeptide chain folds into a unique three-dimensional structure has largely remained enigmatic. Useful knowledge has been gained on the plausible structural events that occur during the folding of proteins from their denatured state(s)^2,3^. Stable intermediate state(s) that populate in the equilibrium/kinetic folding/unfolding pathways of several proteins have been characterized^4,5^. It is widely believed that the folding polypeptide is partitioned between productive intra-chain interactions leading to the formation of the native conformation and non-productive inter-chain interactions that result in misfolding and consequently aggregation of the protein^6,7^. Aggregates formed can range from amorphous structures without order to highly structured fibrils as observed in dilapidating amyloid diseases such as Alzheimer’s disease, Parkinson’s disease, serum amyloid-A (SAA), and type-II diabetes^8–10^. In several cases, the propensity to aggregate is attributed to inter-chain interactions between solvent-exposed hydrophobic surfaces present in obligatory and non-obligatory partially structured intermediates that populate the kinetic/equilibrium folding/unfolding pathways of proteins^11^. In this context, understanding the structural determinants governing protein aggregation is critical for the rational design of drugs against the multitude of amyloid diseases^12^.

The folding/unfolding pathways of single and multidomain proteins have been extensively studied^13,14^. However, most of these proteins are predominantly helical^15^. Studies examining the kinetics of all helical proteins such as myoglobin have indicated that α-helical proteins refold from their denatured state(s) very rapidly on the millisecond timescale^16^. The higher rate of refolding of all-helical single domain proteins is believed to be due to the ease of formation of intra-strand backbone hydrogen bonds in helical conformations. Interestingly, very little information exists on the kinetics of folding of all beta-sheet proteins. A detailed knowledge of the kinetic events in the folding of all-beta-sheet proteins will likely provide valuable information on the structural forces that trigger aggregation which mostly involves organization of the polypeptide backbone into an array of beta-sheets^17,18^.

**Figure 1:**
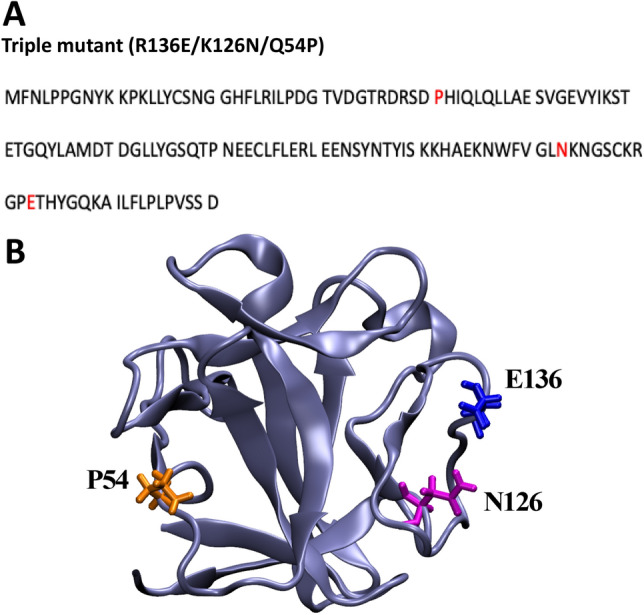
Amino acid sequence of the triple variant of hFGF1. The residues highlighted in red represent the mutated residues in wtFGF1 (Q54P, K126N, and R136E) (**A**). Cartoon representation of hFGF1 structure (PDB ID: 1RG8) showing the mutation sites (**B**).

Human acidic fibroblast growth factor (hFGF1) is a ~ 16 kDa protein that aids in the regulation of key cellular processes such as cell proliferation, angiogenesis, tumor metastasis, and wound healing^19–23^. hFGF1 has a low half -life *in vivo*^24^. Interestingly, a significant population of hFGF1 has been shown to exist in partially unfolded states at temperatures which are marginally higher than the physiological temperature^25^. Previous studies have demonstrated that the denaturant-induced equilibrium unfolding/refolding of hFGF1 does not occur via a two-state mechanism. Samuel et al*.,* showed that GdnHCl-induced equilibrium unfolding of hFGF1 proceeds through the formation of an obligatory intermediate^26^. Similarly, Alsenaidy et al*.,* have shown that hFGF1 is highly prone to aggregation when subjected to heat beyond its T_*m*_ (< 40 °C) or when subjected to minor changes in pH^27^. Blaber and coworkers elucidated that the thermal unfolding of hFGF1 can only be reversed by the addition of low concentrations of GdnHCl^28^. It is believed that addition of low concentrations of the denaturant potentially destabilizes the stable obligatory intermediate that accumulates in the GdnHCl-induced equilibrium unfolding pathway of hFGF1^26^. The propensity of hFGF1 to heavily aggregate has been a significant impediment in realizing its strong wound healing potential. In this context, there has been an increased interest in devising new wound healing formulations that could stabilize hFGF1. Therefore, in this study, we have designed a hFGF1 triple variant (TM) (Q54P/K126N/R136E) that not only exhibits significantly higher thermal stability but also exhibits reversible thermal unfolding by resisting the formation of aggregates (Fig. 1). We believe that the findings of this study shed valuable insights into the interplay of structural forces that confer reversible unfolding behavior to the protein and also provides useful clues for the rational design of a hFGF1-based therapeutics to manage chronic wounds.

## Materials and methods

DNA plasmid isolation kits were purchased from Qiagen, USA and Quikchange II XL mutagenesis kits were obtained from Agilent. Competent cells (DH5α and BL-21(DE3)) were sourced from Novagen Inc., USA. Lysogeny broth (LB) was obtained from EMD Millipore, USA. Heparin sepharose was obtained from GE Healthcare, USA. VWR Scientific Inc, USA was the supplier for all buffer components including Na_2_HPO4, NaH_2_PO4 and NaCl. NIH 3T3 cells were obtained from ATCC and all the cell culture reagents including, DMEM media, fetal bovine serum (FBS) and penicillin–streptomycin were purchased from Thermo Fisher Scientific (Waltham, MA).

### Construction and availability of pure wtFGF1 and hFGF1 variant (s)

For all site-directed mutagenesis experiments, a truncated form of the hFGF1 gene (residue number, 15-154) was inserted into a pET20b expression vector. Primers were designed using an Agilent primer design program and were ordered from IDT DNA Inc., USA. Site-directed mutagenesis (SDM) was performed using a QuikChange lightning kit followed by polymerase chain reaction (PCR) as per the protocol provided by the vendor (Agilent Technologies). The plasmid was then transformed into XL-gold competent cells and plasmid sequencing was carried out by the DNA core sequencing facility at the University of Arkansas Medical Science (UAMS). Each hFGF1 variant was overexpressed in BL-21(pLysS) *Escherichia coli* cells cultured in lysogeny broth (LB) at 37 °C with agitation at 250 rpm. After overexpression, bacterial cells were lysed by ultra-sonication and the released proteins were separated from the cell debris by centrifugation for 20 min at 19,000 rpm. All the hFGF1 variant proteins were then purified on a heparin-sepharose column using a stepwise salt gradient (100–1500 mM NaCl) in 10 mM sodium phosphate buffer (pH 7.2). SDS-PAGE was used to determine the purity of the protein. The protein bands were visualized by staining the gels with Coomassie brilliant blue and the protein concentrations were determined by Bradford assay using a Hitachi F-2500 fluorimeter.

### Circular dichroism spectroscopy

Far-UV CD measurements of wtFGF1 and hFGF1 variants were performed on a JASCO-1500 spectropolarimeter using a quartz cell of 1 mm path length. Each spectrum was recorded as an average of 3 scans and the wavelength range used was from 190 to 250 nm^19,20^. The concentration of the protein used was 30–35 μM in 10 mM sodium phosphate buffer (pH 7.2) containing 100 mM NaCl at 25 °C. All the data points were baseline corrected and smoothened using the Savitzky-Golay algorithm. The final spectra (smoothened and normalized curves) were obtained after necessary blank corrections with 10 mM sodium phosphate buffer (pH 7.2) containing 100 mM NaCl.

### Fluorescence spectroscopy

All intrinsic fluorescence measurements were performed on a Hitachi F-2500 spectrophotometer at 25 °C and a 10 mm quartz cuvette. The excitation and emission slit widths were each set at 2.5 nm. All the experiments were performed with protein concentration of 30–35 μM in 10 mM sodium phosphate buffer (pH 7.2) containing 100 mM NaCl. wtFGF1 and all the variant proteins were excited at a wavelength of 280 nm and the emission spectra were recorded from 300 to 400 nm. The final spectra were plotted after normalization and smoothening of the curves to eliminate plausible minor variance in the concentration of the proteins.

### Equilibrium unfolding

The thermal unfolding data were acquired using J-1500 spectropolarimeter by simultaneously monitoring changes in fluorescence and ellipticity changes (at 222 nm). However, to avoid redundancy of information, we did not report the ellipticity changes that occur during thermal unfolding of hFGF1, and the designed variants, in this manuscript. The urea and GdnHCl unfolding of wtFGF1 and all the hFGF1 variants were performed on a Jasco-1500 spectrophotometer using the fluorescence. Equilibrium unfolding experiments were performed using a protein concentration of 30–35 μM in 10 mM sodium phosphate buffer (pH 7.2) containing 100 mM NaCl. Spectra were collected every 5 degrees from 25 to 90 °C and the scan rate was 0.5 °C/min for all the experiments unless stated otherwise. Each set of data was fit using excel graphing tools. The proteins were excited at a wavelength of 280 nm and the emission spectra were recorded from 300 to 400 nm and the fraction unfolded was plotted as a function of temperature. The denaturation temperature (T_*m*_) and concentration (C_*m*_) were determined as the temperature and concentration at which 50% of the protein population was denatured. Urea and GdnHCl-induced equilibrium unfolding experiments were conducted at a protein concentration of 30–35 μM Urea and GdnHCl was titrated in consistent volumes into the sample up to a concentration of 8 M and 6 M, respectively. The preincubation time between each titration was 5 min. Protein unfolding was monitored by fluorescence and the fraction unfolded (F_d_) was calculated as per the method reported by Bhuyan et al.^29^. Spectra were collected as an average of 3 scans.

### Nuclear magnetic spectroscopy

^1^H-^15^N HSQC experiments were conducted on a Bruker Avance DMX-700 MHz spectrometer equipped with a 5 mm inverse cryoprobe at 25 °C. wtFGF1 and the Q54P/K126N/R136E-TM were grown in M9 medium with ^15^NH_4_Cl used as the sole nitrogen source. For the heat treated Q54P/K126N/R136E-TM NMR sample, the protein was heated to 75 °C using the water bath for 3 min and then cooled down at room temperature for 5 min. The sample was then centrifuged to remove any visible aggregates. ^15^N labeled protein samples (1 mM) were prepared in 90% H_2_O + 10% D_2_O solution containing 10 mM sodium phosphate buffer (pH 7.2) containing 100 mM NaCl and 25 mM (NH_4_)_2_SO_4_. Spectra were recorded using 48 scans and 256 data points in XY dimension. Data were analyzed using XWINNMR 3.5 software supplied by Bruker Biospin Inc., USA.

### Cell proliferation activity

Cell proliferation assay was performed on NIH-3T3 fibroblast cells (obtained from ATCC (Manassas, VA)) using the method reported by Julie *et al*^30^. The experiment was conducted in triplicate (n = 3). Cells were maintained at 37 °C with 5% CO in complete media consisting of Dulbecco's Modified Essential Medium (DMEM) supplemented with 10% FBS, L-glutamine, and 1% penicillin. At 80–90% confluency, cells were transferred to incomplete media (complete media without 10% FBS). The mitogenic activity of wtFGF1 and the variants was estimated by incubating the cells at varying concentrations (0, 0.4, 2, 10, and 50 ng/mL) of hFGF1. After 24 h of incubation, the number of 3T3 cells were quantified using CellTiter-Glo (Promega, Madison, WI) cell proliferation assay^20^.

### All-atom equilibrium MD simulations

An all-atom equilibrium MD simulation of the triple variant (Q54P, K126N, R136E) was performed based on the X-ray crystal structure of the hFGF1 monomer (PDB ID: 1RG8 – Resolution: 1.1 angstroms)^31^. CHARMM-GUI^32,33^ was used to generate the initial simulation model. Residues 12–137 from the crystal structure correspond to residues 26–151 in the experimental sequence. The first 12 residues were not used experimentally because they are part of a random coil. The heparin-binding region (HBR) spans residues 126–142 (in the experimental sequence). The initial part of the simulation was performed with the NAMD 2.13 simulation package and the CHARMM36 all-atom additive force field^34,35^. The input files were generated using CHARMM-GUI’s QuickMD Simulator plugin^33^. The model was solvated in a rectangular TIP3P water box and 150 mM of NaCl ions were inserted into the system using the Monte-Carlo ion placing method^33^. The system consisted of approximately 25,000 atoms. Initially, we energy-minimized the system for 10,000 steps using the conjugate gradient algorithm^36^. Then, the system was relaxed by releasing the restraints in a stepwise manner (for a total of ∼1 ns) using the standard CHARMM-GUI equilibration protocol^32,33^. The initial relaxation was performed in an NVT ensemble while the rest of the simulation was performed in an NPT ensemble at 300 K using a Langevin integrator with a time step of 2 fs and damping coefficient of 0.5 ps^−1^. The pressure was maintained at 1 atm using the Nosé–Hoover Langevin piston method^36,37^. The smoothed cut-off distance for non-bonded interactions was set to 10–12 Å, and long-range electrostatic interactions were computed with the particle mesh Ewald (PME) method^38^. The initial equilibration run lasted 15 ns. The production run was then extended on the Anton2 supercomputer for 4.8 microseconds, with a timestep of 2.5 fs. The pressure was maintained at 1 atm semi-isotropically, using the MTK barostat, while the temperature was maintained at 300 K, using the Nosé–Hoover thermostat. The long-range electrostatic interactions were computed using the fast Fourier transform (FFT) method^39^. Conformations were collected every 240 picoseconds. Data from an all-atom equilibrium MD simulation of wildtype hFGF1 that we had performed previously using identical conditions^40^, was compared with data from the triple variant simulation described above.

The RMSD trajectory tool of VMD was used to calculate the RMSD and C_α_ atoms were considered for these calculations^41^. For RMSD calculations, we aligned the region of interest against its own initial configuration and calculated RMSD with respect to this configuration. RMSF of individual residues was calculated using the C_*α*_ atoms by aligning the trajectory against the crystal structure. The salt bridge plugin of VMD was used to calculate the distance between the two salt bridge residues over the course of the simulation, which is the distance between the oxygen atom of the participating acidic residue and the nitrogen atom of the basic residue. The VMD HBond plugin was used for hydrogen bond analysis^41^. Salt bridges were defined as interactions with an oxygen–nitrogen cut-off distance below 4 angstroms for at least 50% of the total simulation time^41^. Hydrogen bonds were defined as interactions between two residues with a hydrogen donor and a hydrogen acceptor with an angle cutoff of 35 degrees between the donor, hydrogen, and acceptor and a donor–acceptor distance cutoff of 4 angstroms. A stable hydrogen bond was defined as a hydrogen bond with an occupancy of 50% or more over the course of the simulation. Solvent Accessible Surface Area (SASA) was calculated using the internal SASA measurement method of VMD^41^.

## Results and discussion

### Rationale for the designed mutations

As mentioned previously, hFGF1 is an all beta sheet protein with 12 beta strands organized into a β-trefoil structure. The flexible heparin-binding pocket (located between β-10 and 12) contains a high density of positively charged residues (Fig. 1). We recently demonstrated that a charge reversal mutation (R136E) in the heparin-binding pocket (HBP) marginally decreases the heparin-binding affinity but enhances the cell proliferation activity of hFGF1^19^. hFGF1 is known to be inherently unstable molecule at temperatures just above the physiological temperature^20^. It is believed that the instability of hFGF1 largely stems from the charge repulsions between the closely placed positively charged residues in the HBP^19^. In this context, introduction of a negative charge in the HBP is postulated to provide a counter-ion effect and consequently stabilize hFGF1. K126 is located in the periphery of the HBP and has been shown to contribute significantly to the heparin-binding affinity^19^. Therefore, we expect that neutralization of the charge via K126N mutation would not only decrease the heparin-binding affinity but also increase the stability of hFGF1. Contribution of two negative charges in the cationic heparin-binding pocket is expected to significantly stabilize hFGF1. Thus, introduction of Q54P, K126N, and R136E mutations could plausibly induce a stable conformation by improving the interactions (salt bridges) in the protein core and stabilizing the protein against temperature and chemical denaturants. Q54 is lodged in the loop connecting β-3 and 4. This β-turn falls under the category of type I or type IV turns. In either of these β-turns, proline is 4.3 times more favored than Gln^42^. In this context, Q54P is expected to make the molecule more compact and consequently stabilize additional interactions in the protein core. Zakrzewska et al*.,* showed that Q54P mutation significantly increases the stability and cell proliferation activity of hFGF1^42^. In addition, secondary structural analyses of wtFGF1 also revealed the presence of short 3_10_-helices. Again, Pro is a favored amino acid in 3_10_-helices. Matthews et al*.,* and Mateos et al*.,* observed that proline mutations decrease the conformational entropy of the unfolded state of proteins^43,44^. Thus, introduction of Q54P, K126N, and R136E plausibly aid in minimizing the exposure of hydrophobic regions to the surface and restricts the conformational fluctuations occurring in the HBR, thereby leading to refolding and higher stability of wtFGF1. In this context, seven (R136E, K126N, Q54P, Q54P/R136E, Q54P/K126N, K126N/R136E and Q54P/K126N/R136E) variants were designed to specifically delineate their effects on the structure, stability, and cell proliferation activity of hFGF1.

### Mutations do not alter the structure of hFGF1

**Figure 2:**
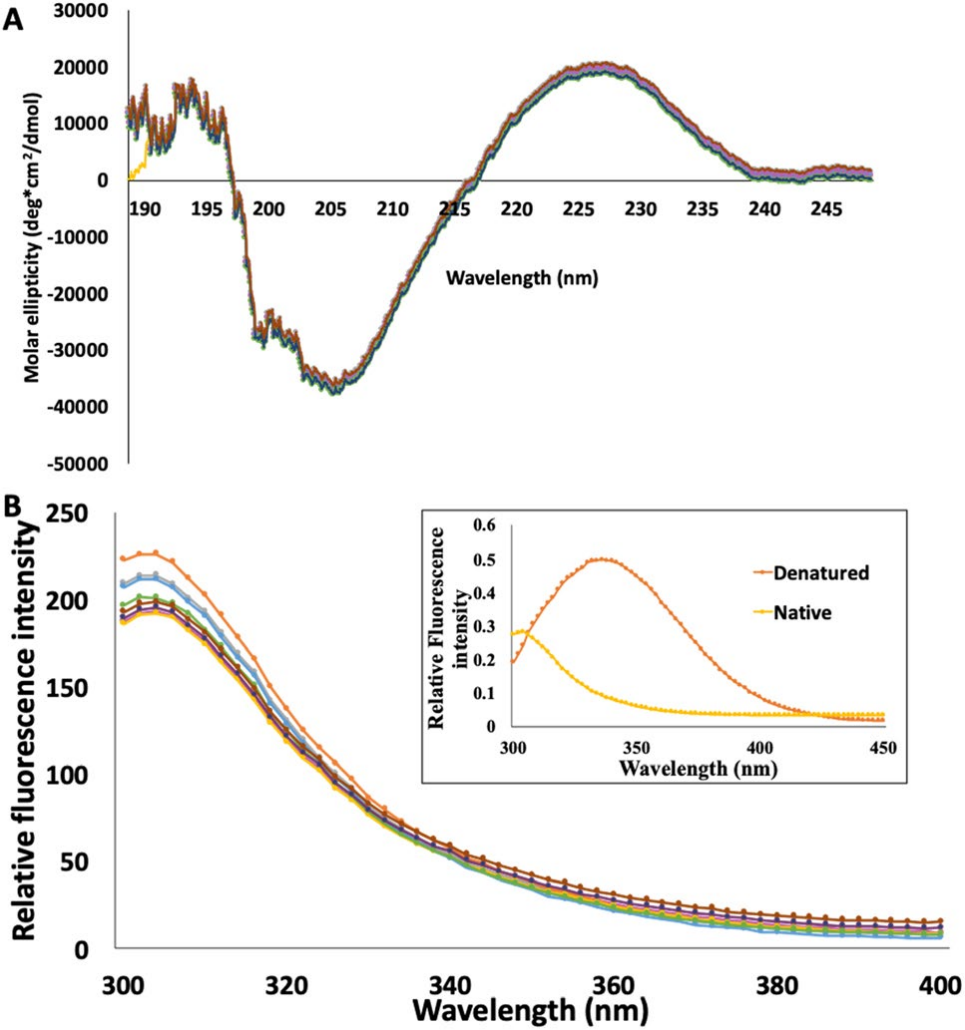
Overlay of the far-UV CD spectra of wtFGF1 and the designed variants (**A**). Overlay of the fluorescence spectra showing the similarity in the tertiary structure of wtFGF1 and the designed variants (**B**). The inset figure represents a cartoon representation of the intrinsic fluorescence spectra of hFGF1 in its native and denatured state(s). Concentration of protein used was 30–35 μM in 10 mM phosphate buffer, pH 7.2 containing 100 mM NaCl. wtFGF1 (pink), R136E (orange), K126N (gray), Q54P (yellow), K126N/R136E (purple), Q54P/R136E (green), Q54P/K126N (blue), and Q54P/K126N/R136E (brown).

wtFGF1 and the designed variants (R136E, K126N, Q54P, Q54P/R136E, Q54P/K126N, K126N/R136E and Q54P/K126N/R136E) were purified to homogeneity using affinity and gel filtration column chromatography (Supplementary Fig. S1). It is important to verify if the introduction of mutations significantly perturbed the structure of hFGF1. In this context, we used far-UV circular dichroism (CD) spectroscopy and intrinsic fluorescence spectroscopy to monitor the structural changes that could potentially occur as a consequence of the introduced mutations. Far-UV CD spectrum (190–250 nm) of wtFGF1 shows positive and negative ellipticity bands centered at around 228 nm and 205 nm, respectively (Fig. 2A). These structural features are consistent with the β-trefoil structure of hFGF1. Interestingly, the far-UV CD spectra of the hFGF1 variants superimpose quite well with that of wtFGF1 suggesting that the secondary structure of the protein is not perturbed due to the designed mutations.

hFGF1 contains eight tyrosine residues and a lone tryptophan residue (Trp121, Fig. 1A). Intrinsic fluorescence of a single tryptophan residue in the native conformation is significantly quenched by the surrounding amine groups of lysine and proline residues^19^. As a consequence, intrinsic fluorescence spectrum of wtFGF1 in its native state shows an emission maximum at 308 nm corresponding to the tyrosine fluorescence (Fig. 2B). However, the quenching effect of tryptophan is completely relieved in the unfolded state(s) and shows a characteristic emission maximum at around 350 nm (Fig. 2B, inset). Therefore, monitoring changes in the intrinsic fluorescence provides a reliable measure of the tertiary structural changes that could plausibly occur in the protein. Overlay of the intrinsic fluorescence spectra of the hFGF1 variants revealed little or no significant differences indicating that the tertiary structural contacts in the protein mostly remain unperturbed due to introduction of the mutations.

### Effect (s) of mutation on the thermal stability of hFGF1 variants

As mentioned previously, hFGF1 is an unstable molecule [T_*m* (apparent)_ ~ 41 °C, T_*m*_ is the temperature at which 50% of the protein population exists in denatured state(s)]^28^ (Table 1). wtFGF1 is known to aggregate when subjected to thermal unfolding at temperatures above its T_*m* (apparent)_^45^. In addition, thermal unfolding of hFGF1 is known to be irreversible and consequently the calculated T_*m* (apparent)_ is only a qualitative measure of the thermal stability of the protein^45^. More recently, Longo and coworkers showed that the thermal unfolding of de novo designed Phifoil protein (bearing structural resemblance to FGF1) can be reversed at a critical concentration of 0.22 μM^45^. However, reversible thermal unfolding of wt-hFGF1 still required the presence of low concentrations (0.7–1 M) of guanidinium hydrochloride^28^. The low thermal stability and the problems associated with irreversibility of the thermal unfolding have significantly impeded the development of hFGF1 based therapeutics for chronic wound care. In this context, we investigate the stability of wtFGF1 and the variants by monitoring the changes in the intrinsic fluorescence intensity ratio at 308 nm and 350 nm (Supplementary Fig. S2).

**Table 1:** Comparison of the stability of wtFGF1 and the designed variants.

Proteins	T_*m* (apparent)_ value (°C)	C_*m*_ value (M Urea)	C_*m*_ value (M GdnHCl)
wtFGF1	41.5 ± 0.1	1.9 ± 0.4	1.0 ± 0.8
R136E	52 ± 0.2	2.25 ± 0.1	1.82 ± 0.4
K126N	54 ± 0.1	2.3 ± 0.3	1.87 ± 0.3
Q54P	43 ± 0.1	1.52 ± 0.3	1.44 ± 0.5
Q54P/R136E	53 ± 0.1	1.53 ± 0.2	1.53 ± 0.6
Q54P/K126N	56 ± 0.5	1.55 ± 0.1	1.43 ± 0.1
K126N/R136E	49 ± 0.1	1.70 ± 0.1	1.74 ± 0.2
Q54P/K126N/R136E	60 ± 0.1	3.97 ± 0.1	2.35 ± 0.1

**Figure 3:**
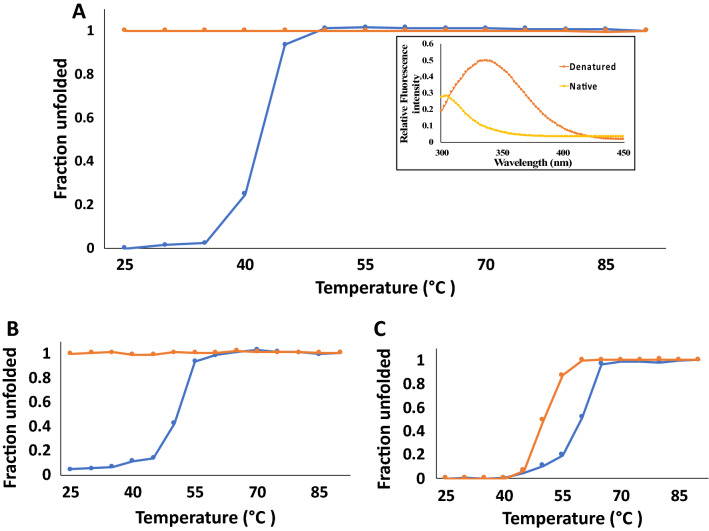
Thermal unfolding and refolding curves of wtFGF1 (**A**; unfolding—blue, refolding—orange), R136E hFGF1 variant (**B**; unfolding—blue, refolding—orange), and TM-variant of hFGF1 (**C**; unfolding—blue, refolding—orange). The thermal unfolding and refolding of wtFGF1 and its variants were monitored by changes in the ratio of intrinsic fluorescence intensities at 308 nm to 350 nm. Insert figure in (**A**) depicts the intrinsic fluorescence spectra of hFGF1 in its native and denatured states. Concentration of protein used was 30–35 μM in 10 mM phosphate buffer, pH 7.2 containing 100 mM NaCl.

Thermal unfolding data suggests that wtFGF1 (T_*m* (apparent)_—41 °C) exhibits the lowest thermal stability (Table 1). Q54P mutation appears to have a marginal effect (T_*m* (apparent)_ =  ~ 43 °C, Fig. 3, Table 1) on the stability of the protein. Interestingly, single point mutations (K126N and R136E) in the heparin-binding pocket appear to have a more profound effect (~ 10–12 °C) on the thermal stability of hFGF1 (Table 1 and Fig. 3). These results suggest that the charge repulsions in the cationic HBP are primarily responsible for the inherent instability of hFGF1. K126N mutation not only decreases the repulsions between cationic residues in the heparin-binding pocket but also appears to facilitate forging of a backbone to side-chain hydrogen bonding between N126 and S130. It is believed that introduction of a negative charge via the R136E mutation induces a counter-ion effect in the HBP which partially screens the charge repulsions. Surprisingly, the double mutation (K126N/R136E) in the HBP shows a noticeably lower (T_*m* (apparent)_ ~ 49 °C, Table 1 and Supplementary Fig. S3) stability than the corresponding individual single-point mutations (K126N and R136E).

Although it is not clear why simultaneous replacement of K126 and R136 causes the observed decrease in stability, this is interesting considering the fact that these individual mutations distinctly stabilize the protein. It appears that the individual stabilizing forces that come into play in the heparin-binding pocket (due to K126N and R136E mutations) mutually weaken each other leading to a net decrease in the stability of the protein. On the other hand, K126N and R136E mutations when individually combined with Q54P, to yield the double variants (Q54P/K126N and Q54P/R136E), confer significant stability to wtFGF1 (Supplementary Fig. S3, Table 1). These results once again confirm that maximum effects on the stability of the protein are caused by nullification or reversal of charges in the heparin-binding pocket. Very interestingly, Q54P mutation appears to compensate for the mutually destabilizing effects of K126N and R136E mutations. This is obvious from the high T_*m*(apparent)_ value of the triple variant, Q54P/K126N/R136E. Although Q54P is located remotely from the mutation sites in the HBP, the structural kink introduced by proline appears to be transmitted through a network of interactions to the heparin-binding pocket and consequently enables the two mutations (K126N and R136E) to synergistically produce a stabilizing effect on hFGF1.

### Mutations in the heparin-binding pocket also affect stability of hFGF1 to chemical denaturants

**Figure 4:**
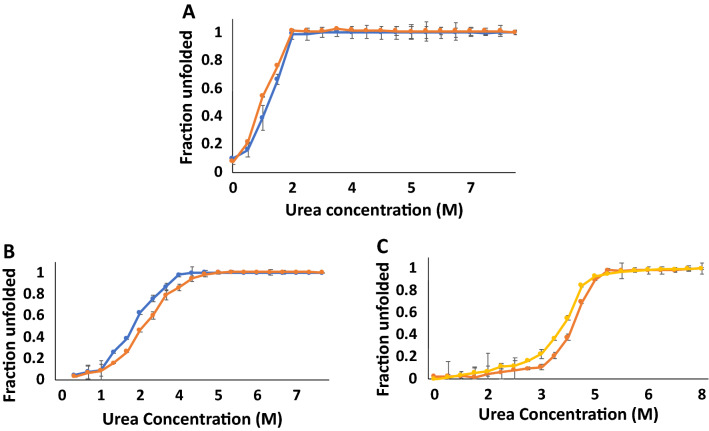
Urea-induced equilibrium unfolding and refolding curves of wtFGF1 (**A**; unfolding—blue, refolding—orange), R136E hFGF1 variant (**B**; unfolding—orange, refolding—blue), and TM-variant of hFGF1 (**C**; unfolding—yellow, refolding—orange). The urea-induced equilibrium unfolding/refolding of wtFGF1 and its variants was monitored by changes in the ratio of intrinsic fluorescence intensities at 308 nm to 350 nm. Concentration of protein used was 30–35 μM in 10 mM phosphate buffer, pH 7.2 containing 100 mM NaCl.

**Figure 5:**
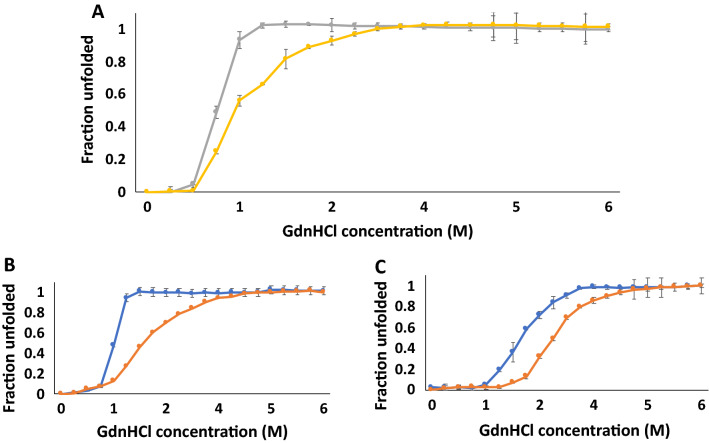
GdnHCl-induced equilibrium unfolding and refolding curves of wtFGF1 (**A**; unfolding—yellow, refolding—gray), R136E hFGF1 variant (**B**; unfolding—orange, refolding—blue), and TM-variant of hFGF1 (**C**; unfolding—orange, refolding—blue). The GdnHCl-induced equilibrium unfolding/refolding of wtFGF1 and its variants was monitored by changes in the ratio of intrinsic fluorescence intensities at 308–350 nm. Concentration of protein used was 30–35 μM in 10 mM phosphate buffer, pH 7.2 containing 100 mM NaCl.

Chemical denaturant-induced isothermic equilibrium unfolding of hFGF1 was examined to study the effects of the mutations on the stability using intrinsic fluorescence spectroscopy (Figs. 4 and 5). Urea-induced equilibrium unfolding of wtFGF1 at pH 7.2 (Fig. 4) shows that it is a relatively unstable molecule [C_*m*_ ~ 1.9 M, C_*m*_ is the concentration of the denaturant wherein 50% of the protein population exists in the denatured state(s)] (Table 1). Similar to the thermal unfolding data, single point mutations (K126N and R136E) in the heparin-binding pocket increase the resistance of the protein to the urea denaturation. However, unlike the thermal denaturation data, urea-induced unfolding data suggests that Q54P does not contribute to the stability of the protein. In fact, the Q54P mutation marginally decreases the C_*m*_ (C_*m*_ ~ 1.5 M). Interestingly, the double variants (Q54P/K126N and Q54P/R136E) which increased the thermal stability of the protein, did not confer extra stability against urea denaturation. In fact, the C_*m*_ (C_*m*_ ~ 1.5 M) values of these double variants were in the similar range to that of wtFGF1. However, the triple variant (Q54P/K126N/R136E) was most resistant to urea denaturation. These results clearly indicate that the interplay of structural forces during thermal unfolding is subtly different from the interplay of structural forces in urea-induced unfolding of the protein. These results also suggest that caution needs to be exercised when designing proteins based on denaturation data obtained from one-set of conditions.

GdnHCl is an ionic denaturant and is known to efficiently disrupt both hydrogen bonds and electrostatic interactions in proteins. We examined the GdnHCl-induced equilibrium unfolding of hFGF1 to understand if the trends in the relative stability of the variants are similar to those observed in the presence of a neutral denaturant, urea. GdnHCl-induced equilibrium unfolding of hFGF1 was studied previously under different buffer conditions and a stable obligate intermediate was found to be populated at around 0.96 M GdnHCl^31^. The partially folded obligatory intermediate was shown to have characteristics resembling that of a molten-globule-like state. In this study, we find that the C_*m*_ of wtFGF1 is 1 M (Table 1, Fig. 5) and is very close to the one reported earlier^46^. All the hFGF1 variants investigated herein showed higher stability than wtFGF1. This trend in the C_*m*_ values is slightly different from those calculated from the urea-induced unfolding profiles. Interestingly, the double mutants (Q54P/K126N, Q54P/R136E, and K126N/R136E) seem to exhibit slightly lower stability than the single point variants (Table 1). These results once again emphasize that the relative stabilities of hFGF1 appear to vary depending on the nature of the denaturant used. However, one aspect that can be consistently inferred from all the denaturation experiments is the extremely high stability of Q54P/K126N/R136E.

### Reversibility of the thermal unfolding of hFGF1

Thermal unfolding of hFGF1 is irreversible. Heating hFGF1 beyond its T_*m* (apparent)_ causes aggregation of the protein. There has been increased interest in designing variants of hFGF1 which show increased stability and enhanced cell proliferation activity. In this context, we examined the reversibility of the temperature-induced unfolding of hFGF1 and its variants by monitoring intrinsic fluorescence changes at 308 nm and 350 nm. wtFGF1 completely unfolds at temperatures beyond 50 °C^19–21,25^. Refolding of the protein was attempted by slow cooling from 85 to 25 °C and the results clearly show that wtFGF1 and the hFGF1 variants remained in the unfolded state(s) (Fig. 3). In fact, we performed the thermal unfolding of wtFGF1 at a lower protein concentration (12.5 μM) with a temperature gradient of 1 °C and at a thermal unfolding rate of 0.25 °C/min, but still found that the temperature-induced equilibrium unfolding is irreversible (Supplementary Fig. S4). This is obvious from the low 308/350 nm intrinsic ratio. Interestingly, heat-induced denaturation of the triple variant is completely reversible. Intrinsic fluorescence spectrum of the refolded protein at 25 °C overlaid quite well with the protein that was subjected to thermal denaturation at 75 °C (Supplementary Fig. S5).

^1^H-^15^ N HSQC spectrum provides atomic level information on the backbone conformation of proteins. Each crosspeak in the spectrum represents an amino acid in a particular backbone conformation. In this context, ^1^H-^15^N HSQC spectra of the triple variant was acquired before heat treatment and after refolding upon cooling. Superimposition of the ^1^H-^15^N HSQC spectra shows that most of the crosspeaks overlap well suggesting that the triple variant is capable of refolding back to its native conformation from the heat denatured state(s) (Supplementary Fig. S6).

**Figure 6:**
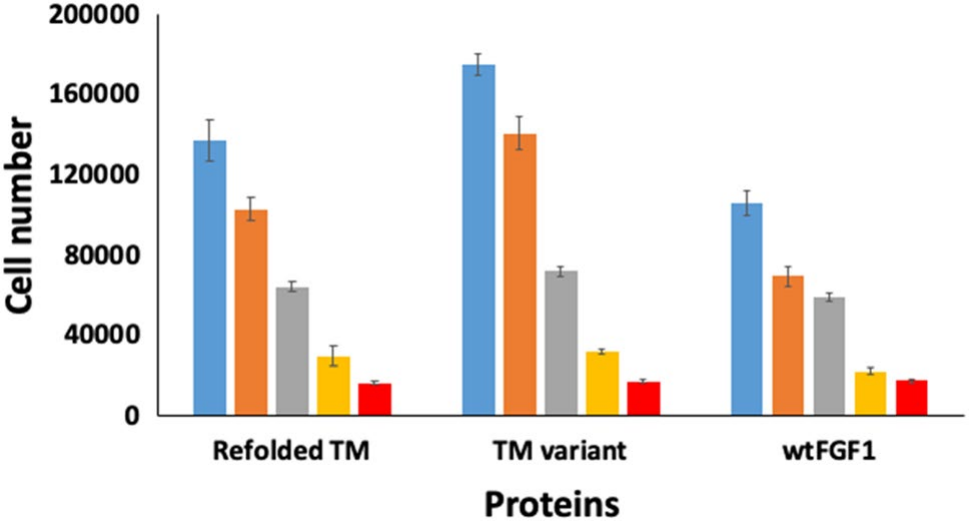
Cell proliferation activity of the wtFGF1 and the triple variant (with and without heat treatment). 50 ng/mL (blue), 10 ng/mL (orange), 2 ng/mL (gray), 0.4 ng/mL (yellow), and 0 ng/mL (red).

hFGF1 is a mitogen and exhibits potent cell proliferation activity. Therefore, the effect(s) of the untreated and refolded triple variant forms on the proliferation of NIH3T3 cells, were compared to examine whether the refolded protein is biologically active. As expected, wtFGF1 promotes proliferation of NIH3T3 cells and the number of cells almost tripled in comparision to the control cells (Fig. 6). Interestingly, the triple variant exhibits higher cell proliferation activity than wtFGF1, which is mostly consistent with its higher structural stability. Refolded triple variant is also biologically active albeit with a lower potency (Fig. 6). The moderlately lower activity exhibited by the refolded triple variant could be either due to lower stability of the refolded protein in the cell culture medium or due to the presence of a small population of biologically inactive soluble oligomers. However, it should be mentioned that no visible aggregates were detected in the refolded triple variant. To our knowledge, this is the only hFGF1 variant that has been unquivocally shown to refold to its unique biologically active conformation from its heat denatured state(s).

### hFGF1 triple variant exhibits hysteresis during refolding from its heat denatured state(s)

Thermal unfolding of the triple variant is reversible but a closer look at the thermal unfolding/refolding curves shows that they do not superimpose well (Supplementary Fig. S7). T_*m* (apparent)_ values characterizing the unfolding (60 ± 0.07 °C) and refolding (50 ± 0.09 °C) processes do not match. Typically, if the structural transitions that occur during heat-induced denaturation and renaturation from the heat denatured state(s) to the native conformation are the same, then one would expect the profiles to superimpose quite well. The mismatch in the T_*m* (apparent)_ values, characterizing the thermal unfolding and refolding processes, is clearly indicative of hysteresis. Observation of hysteresis is uncommon but not unprecedented^47^. Hysteresis observed in the multidomain proteins is largely attributed to the disparities in the folding transition pathways, which probably stem from the fact that unfolding of these proteins is governed by domain transition whereas refolding events occur more cooperatively^48–53^. However, hysteresis in single domain proteins such as collagen is believed to be due to mismatch in the structural events that occur in unfolding-refolding of the protein. Refolding of collagen is believed to occur via slow structural rearrangement of the loop regions whereas unfolding has been shown to be more cooperative requiring the disruption of few structural interactions that are important for stability^49^. Another plausible explanation for the observed hysteresis in the thermal unfolding-refolding of the triple variant may be ascribed to slow *cis–trans* isomerization of the proline (Q54P). Verification of this proposal would require indepth site-directed mutation studies which are beyond the scope of this study. In addition, in some cases hysteresis has also been attributed to transient association between protein molecules during the process of protein folding/unfolding^53^. As protein association is a multimolecular reaction, the hysteresis behavior ascribed to protein association should be dependent on the protein concentration. In this context, we compared the thermal unfolding of the triple variant at two different protein concentrations (33 μM and 12.5 μM). Thermal unfolding/refolding curves obtained at these concentrations were nearly identical, suggesting that the hysteresis phenomenon observed in the triple variant is not related to protein association events (Supplementary Figs. S7A and S7C). In some cases, hysteresis, observed during thermal unfolding-refolding, is believed to be due to steep temperature gradients used during the renaturation process^51^. To verify this aspect, we examined thermal unfolding/refolding of the triple variant under two temperature gradient conditions, 1 °C and 2 °C temperature interval (Supplementary Figs. S7A and S7B). The results obtained suggest that the hysteresis observed during the thermal unfolding-refolding of the triple variant is independent of the temperature gradients used in the experiment.

Chemical denaturant(s)-induced equilibrium unfolding-refolding of the triple variant was examined to understand whether the hysteresis behavior is dependent on the conditions used for protein unfolding/refolding. Overlay of the urea and GdnHCl-induced equilibrium unfolding/refolding profiles separately shows no signs of hysteresis. C_*m*_ values for the chemical-induced equilibrium unfolding/refolding processes appear to be in agreement within the limits of experimental error (Figs. 4C and 5C). Interestingly, these results appear to suggest that hysteresis behavior depends significantly on the conditions used for protein unfolding/refolding. Hysteresis behavior appears to be discernably controlled by the nature of structural intermediates that populate a particular unfolding pathway. In the theoretical sense, it appears that the roughness of the protein folding funnel/terrain is denaturant-dependent.

### Structural interactions contribute to the stability and reversible thermal unfolding of the triple variant

Structural interactions that contribute to the enhanced stability and reversible thermal unfolding of the triple variant were investigated using two-dimensional NMR spectroscopy. Overlay of the ^1^H-^15^N HSQC spectra of wt-hFGF1 on the designed mutations suggest that there are no major backbone structural changes (Supplementary Fig. S8). It indicates that introduction of mutations induces only a minor shift of the cross peaks corresponding to residues located in the spatial vicinity of the mutation site (K132, G134, and R136). These three residues are located in the heparin-binding pocket. R136 is one of the mutation sites and K132 and G134 are involved in the formation of stronger electrostatic interactions (E136-K132, G134-G85) in the triple variant. G134 is in close proximity to R136 and R133 (located in the center of the HBP). Therefore, Q54P, K126N, and R136E mutations appears to have contributed to the formation of new electrostatic interactions in the HBP. These new interactions could have plausibly increased the compactness of the hFGF1 structure and consequently decreased the flexibility of the heparin-binding loop.

### Electrostatic interactions in the heparin-binding pocket stabilize the triple mutant

**Figure 7:**
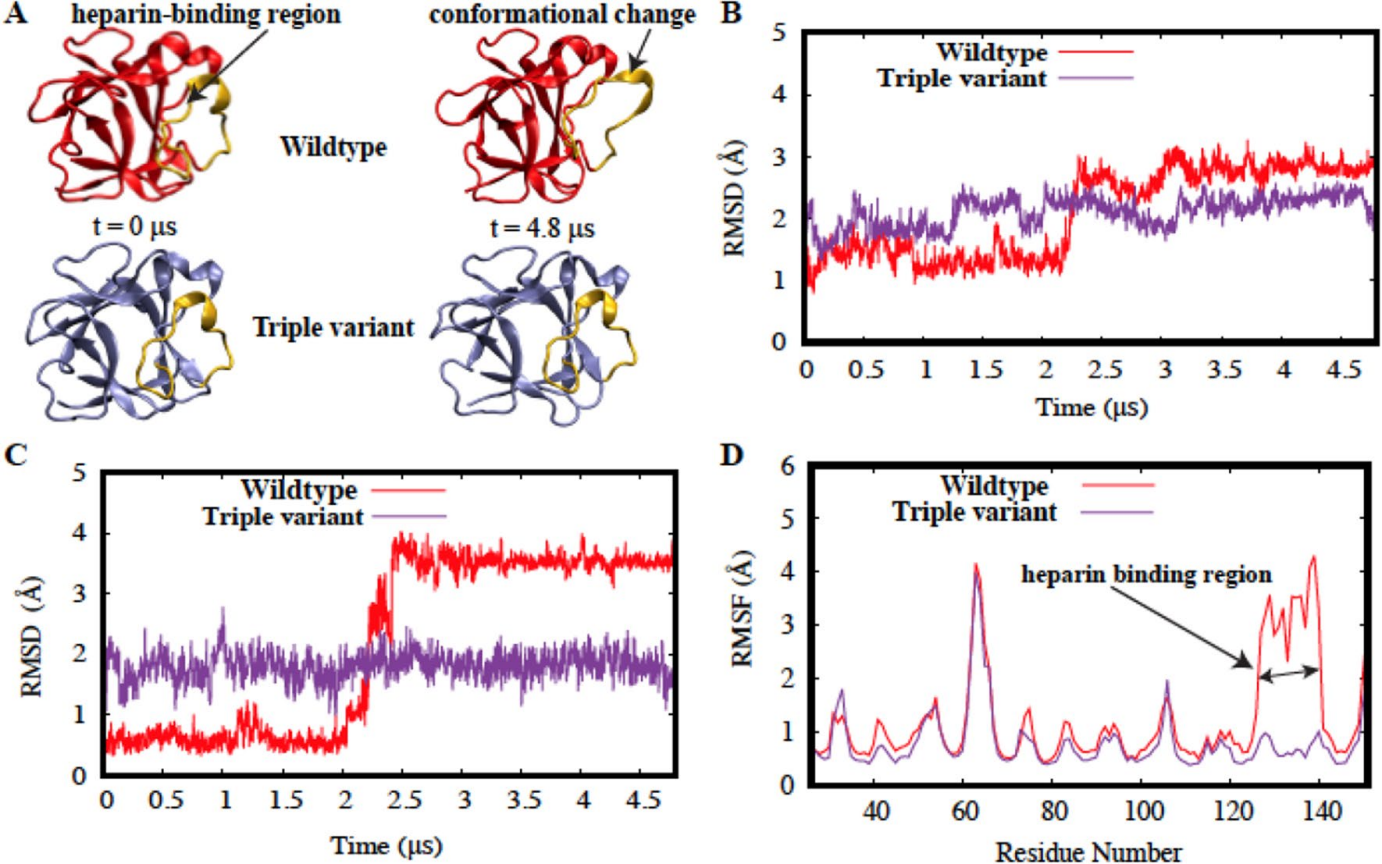
(**A**) Visual representation of wtFGF1 (red) before and after the conformational change. The heparin-binding region (gold) moves away from the beta-trefoil core of the protein. No major conformational change occurs in the triple variant (purple). The heparin-binding region (gold) maintains the same conformation for 4.8 μs. (**B**) RMSD time series for wtFGF1 (red) and the triple variant (purple). (**C**) RMSD time series for the heparin-binding region of wtFGF1 (red) and the triple variant (purple). (**D**) Root mean square fluctuation (RMSF) estimations for the wtFGF1 (red) and the triple variant (purple).

To investigate the structural interactions that potentially determine reversibility of the protein, we have also performed microsecond-level MD simulations of wtFGF1^40^ and the triple variant, based on the X-ray crystal structure of the hFGF1 monomer (PDB ID: 1RG8)^31^. wtFGF1 is fairly stable initially, with RMSD values remaining around 1 Å for approximately 2 µs^40^. However, it undergoes a conformational change quite abruptly after about 2 µs and reaches an RMSD value of nearly 3 Å^40^. When compared with wtFGF1, triple variant does not exhibit any sudden conformational changes and remains relatively stable for 4.8 µs with an RMSD of around 2 Å (Fig. 7B). The differential behavior of wtFGF1 and the triple variant is more clearly reflected in the RMSD time series of the heparin-binding region, wherein wtFGF1 behaves significantly differently from the triple variant (Fig. 7C). Heparin-binding region in wtFGF1 is initially stable below 1 Å but undergoes an abrupt conformational transition (Figs. 7A,C) at around 2 µs and stays at 3.5 Å for the rest of the simulation^40^, whereas the HBR of the triple variant remains stable around 2 Å for 4.8 µs. A visual inspection of the simulation trajectories clearly reveals that the HBR of wtFGF1 moves away from the core of the protein after the 2 µs timepoint^40^, while no major change is observed for the triple variant (Fig. 7A).

These results are further corroborated by the RMSF analysis for wtFGF1^40^ and the triple variant, wherein both systems show similar trends in their fluctuations for different regions with the exception of the heparin-binding region in wtFGF1 (Fig. 7D). Experimental results of intrinsic fluorescence and far-UV CD spectroscopy data are in good agreement with the MD simulation data, suggesting that the mutations do not significantly perturb the beta-trefoil core structure of wtFGF1.

Based on the criteria defined in the methods section, 65 stable hydrogen bonds were identified in wtFGF1^40^. Only one interaction out of 65 involves the heparin-binding region-L145-K142 (84%) which is a backbone-backbone hydrogen bond^40^. All 65 interactions observed in wtFGF1 also occur in the triple variant with similar occupancies. 6 stable hydrogen bonds were observed in the triple variant involving the HBR (Supplementary Table S1), that do not qualify as stable hydrogen bonds in wtFGF1 based on the criteria defined in the methods section. Two of these six interactions involved variant residues (N126 and E136) (Supplementary Table S1 in bold).

Both interactions involving variant residues have very high occupancies. R133-E136 is a strong salt-bridge interaction, indicating that R136E mutation might play a key role in conferring stability to the protein and reversing the unfolding process of wtFGF1 (Supplementary Fig. 9A). A weaker salt bridge involving E136 (E136-K132) (Supplementary Fig. 9B) was also identified. Both these salt-bridges occur only in the triple variant.

Two unique salt-bridges were also identified in wtFGF1–D84-K132 (Supplementary Fig. 9C) and D46-K127 (Supplementary Fig. 9D)^40^. Electrostatic interactions between cationic residues in the heparin-binding region and anionic residues in the beta-trefoil core, help stabilize the HBR after the conformational change that occurs in wtFGF1^40^. D84 of the beta-trefoil core interacts with K132 of the heparin-binding region, while D46 of the beta-trefoil core interacts with K127 of the heparin-binding region^40^. Absence of D84-K132 interaction in the triple variant indicates that K132 interacts more favorably with E136 than with D84. Therefore, R136E mutation potentially causes a transition from a relatively weak long-range interaction to a stronger short-range interaction, thus stabilizing the protein.

Two out of the three mutations (K126N and R136E) in the triple variant are located in the heparin-binding region. RMSD, RMSF and electrostatic interaction analyses suggest that these mutations are the driving force behind the relative conformational stability of the triple variant. These results are further supported by solvent accessible surface area (SASA) calculations for the heparin-binding region. SASA of the wildtype HBR increases significantly after 2 µs^40^ while there is no significant change in the triple variant (Supplementary Fig. S10).

MD simulations reveal that although wtFGF1 is relatively stable within 2 microseconds (which could be misleading if one uses typical sub-microsecond-level simulations), it undergoes a rapid conformational transition after 2 microseconds^40^. Absence of this conformational change in the triple variant indicates that the mutations in the heparin-binding region have led to an increase in stability of the protein. While microsecond-level MD simulations cannot provide a complete characterization of the unfolding and folding process, they do give us unique insights into the conformational dynamics of wildtype and triple variant. The computational data is generally in agreement with the experimental data, showing that these mutations contribute to the structural forces responsible for the reversibility of unfolding of the triple variant.

## Conclusions

In the present study, we show that nullification or charge reversal of amino acids in the heparin-binding pocket of hFGF1 increases the stability without significant perturbation of the three-dimensional structure of the protein. Triple variant (Q54P/K126N/R136E) exhibits ~ 20 °C higher stability than wtFGF1. With the exception of the triple variant, thermal unfolding of wtFGF1 and the variants is irreversible. Two-dimensional NMR and cell proliferation activity data clearly show that the thermally denatured triple variant fully regains its biologically active native conformation upon cooling. Refolding of the triple variant, from its thermally denatured state(s), exhibits a hysteresis behavior with a mismatch in the T_*m* (apparent)_ values of the unfolding-refolding process. In addition, comparison of the thermal-, urea-, and GdnHCl-induced unfolding data suggests that the relative stabilities of hFGF1 and its variants vary depending on the nature of the denaturant used. MD simulation studies reveal that the mutations constrain the flexibility of the heparin-binding region and enhance the stability of hFGF1 by forming a network of stable electrostatic interactions such as R133-E136 and N126-S130. This network of interactions in the triple variant also appears to play a critical role in the reversible thermal unfolding of the protein. To the best of our knowledge, this is the first study wherein a hFGF1 variant with increased stability and reversible protein folding behavior is reported. The findings of this study, in our opinion, can be expected to provide valuable leads for the design of an efficient FGF-based therapy for chronic wound care.

## Supplementary Information

Supplementary Information.
