# Supplementary material for: Characterization of the structural forces governing the reversibility of the thermal unfolding of the human acidic fibroblast growth factor
Source: Sci Rep. 2021 Aug 2;11:15579. doi: 10.1038/s41598-021-95050-2 (PMC8329156; doi:10.1038/s41598-021-95050-2)
Supplement: Supplementary file 1 — Supplementary Information. [file 41598_2021_95050_MOESM1_ESM.pdf]

Characterization of the Structural Forces Governing the Reversibility of the Thermal Unfolding of the Human Acidic Fibroblast Growth Factor

Shilpi Agrawal, Vivek Govind Kumar, Ravi Kumar Gundampati, Mahmoud Moradi and Thallapuranam Krishnaswamy Suresh Kumar**

Department of Chemistry and Biochemistry, University of Arkansas, Fayetteville, Arkansas, United States of America.

SUPPORTING INFORMATION

Supplementary Fig. S1. SDS-PAGE analysis of proteins eluted upon heparin sepharose and gel filtration chromatography. wtFGF1 (Lane – 1), R136E (Lane – 2), K126N (Lane – 3), Q54P (Lane – 4), R136E/K126N (Lane – 5), R136E/Q54P (Lane – 6), and K126N/Q54P (Lane – 7).

Supplementary Fig. S2. Intrinsic fluorescence spectra of thermal unfolding of wtFGF1 (Panel – A) and TM variant of hFGF1 (Panel – B)

Supplementary Fig. S3. Thermal unfolding and refolding curves of wtFGF1 (Panel-**A**; unfolding- blue, refolding- orange), K126N hFGF1 variant (Panel-**B**; unfolding- blue, refolding- orange), Q54P hFGF1 variant (Panel-**C**; unfolding- blue, refolding- orange), Q54P/R136E hFGF1 variant (Panel-**D**; unfolding- blue, refolding- orange), K126N/R136E hFGF1 variant (Panel-**E**; unfolding- blue, refolding- orange), and Q54P/K126N hFGF1 variant (Panel-**F**; unfolding- blue, refolding- orange). The thermal unfolding of wtFGF1 and its variants was monitored by changes in the ratio of intrinsic fluorescence intensities at 308 nm to 350 nm.

Supplementary Fig. S4. Overlay of the intrinsic fluorescence spectrum of the refolded triple variant at 25°C (orange) and the triple variant during thermal denaturation at 75 °C (blue).

Supplementary Fig. S5. Overlay of the ^{1}H–^{15}N HSQC of Q54P-TM without heat treatment (red) and the Q54P-TM after heat treatment (blue) (Panel-A). ^{1}H–^{15}N chemical shift perturbation observed due to the temperature effect (Panel-B). The horizontal line represents the arbitrary threshold above which the ^{1}H–^{15}N chemical shift perturbation(s) was considered as significant. The ^{1}H–^{15}N chemical shift perturbation of individual residues were calculated using the formula, ($\sqrt{[(2\Delta\delta_{NH})^2 + (\Delta\delta_N)^2]}$).

Supplementary Fig. S6. Thermal unfolding and refolding curves of the triple variant at a concentration of 0.5 mg/mL and temperature interval of 5°C (Panel – A; unfolding (blue), refolding (orange)), 0.5 mg/mL and temperature interval of 2°C (Panel – B; unfolding (blue), refolding (orange)), and 0.2 mg/mL and temperature interval of 5°C (Panel – C; unfolding (blue), refolding (orange)).

Supplementary Fig. S7. Overlay of the ^{1}H–^{15}N HSQC of wtFGF1(blue) and the Q54P-TM (red) (Panel-A). ^{1}H–^{15}N chemical shift perturbation observed due to the R136E/K126N/Q54P mutation in hFGF1 (Panel-B). The horizontal line represents the arbitrary threshold above which the ^{1}H–^{15}N chemical shift perturbation(s) was considered as significant. The ^{1}H–^{15}N chemical shift perturbation of individual residues were calculated using the formula, ($\sqrt{[(2\Delta\delta_{NH})^2 + (\Delta\delta_{N})^2]}$).

Supplementary Fig. S8. Panel-A, B: Time series of the E136-R133 (A) and E136-K132 (B) donor-acceptor salt bridge distance in the triple variant structure. Panel- C, D: Time series of the D84-K132 (C) and D46-K127 (D) donor-acceptor salt bridge distances in wtFGF1 (red) and the triple variant (purple).

Supplementary Fig. S9. Comparison of the SASA time series of the heparin-binding region of wtFGF1 and the triple variant.

Supplementary Table S1. Electrostatic interactions involved in the heparin-binding region in the triple variant**.**

Supplementary Fig. S1.

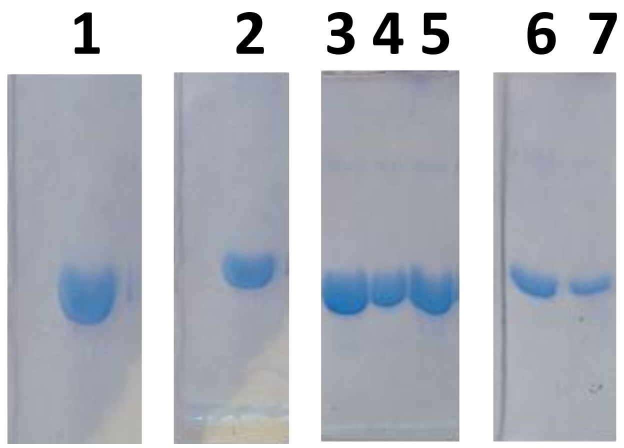

Supplementary Fig. S2.

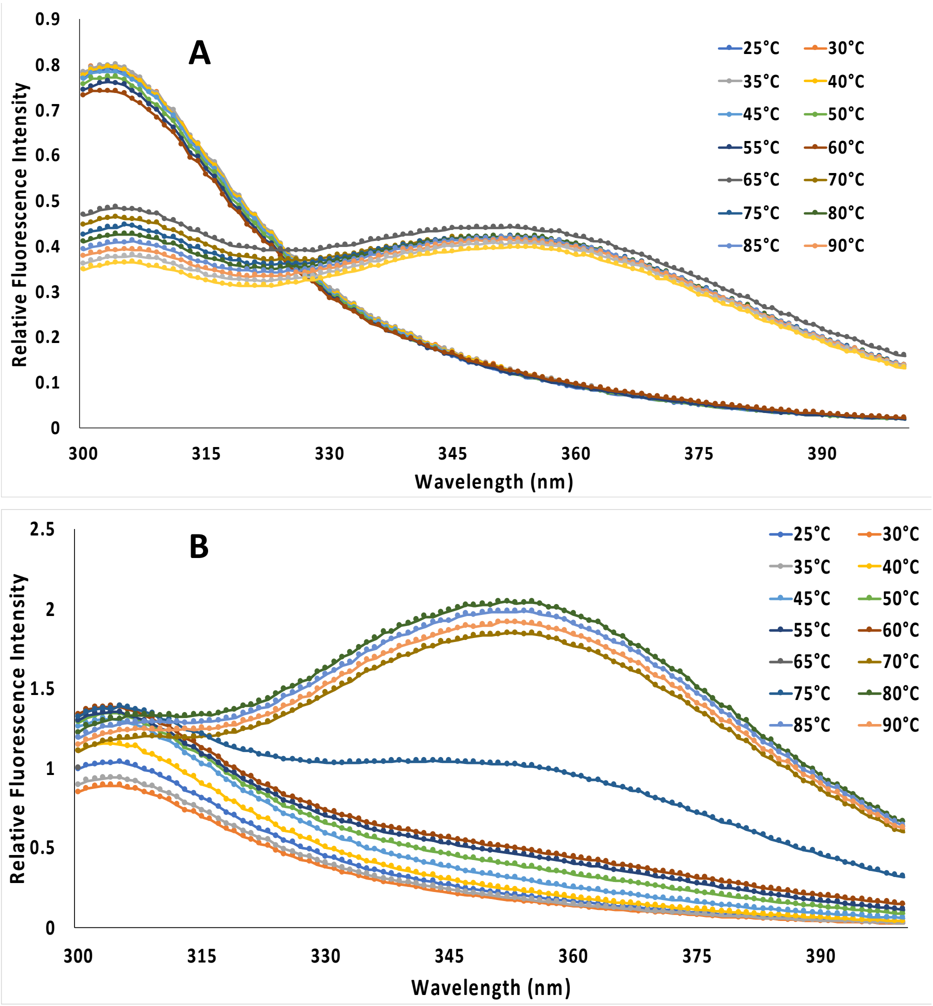

Supplementary Fig. S3.

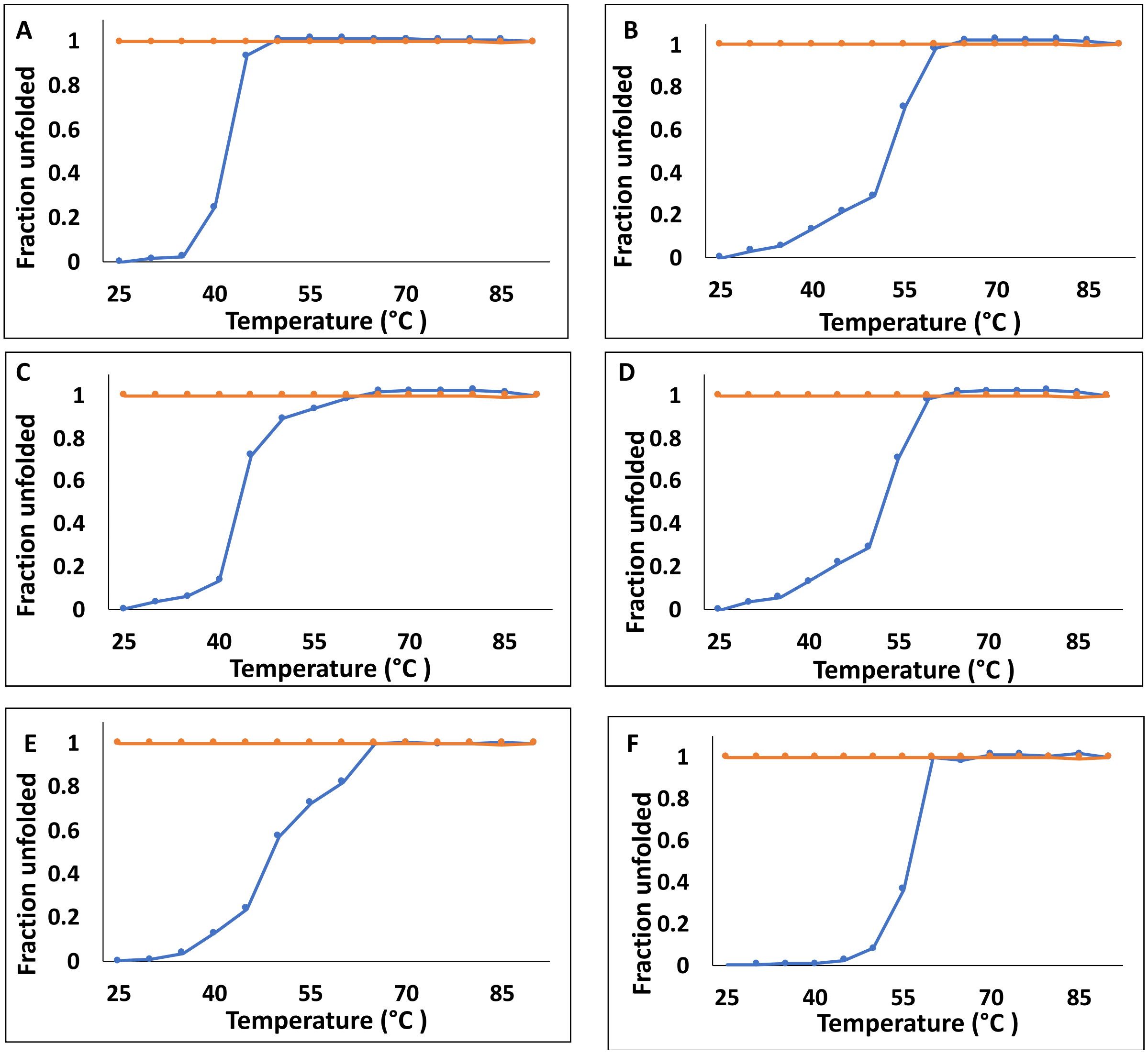

Supplementary Fig. S4.

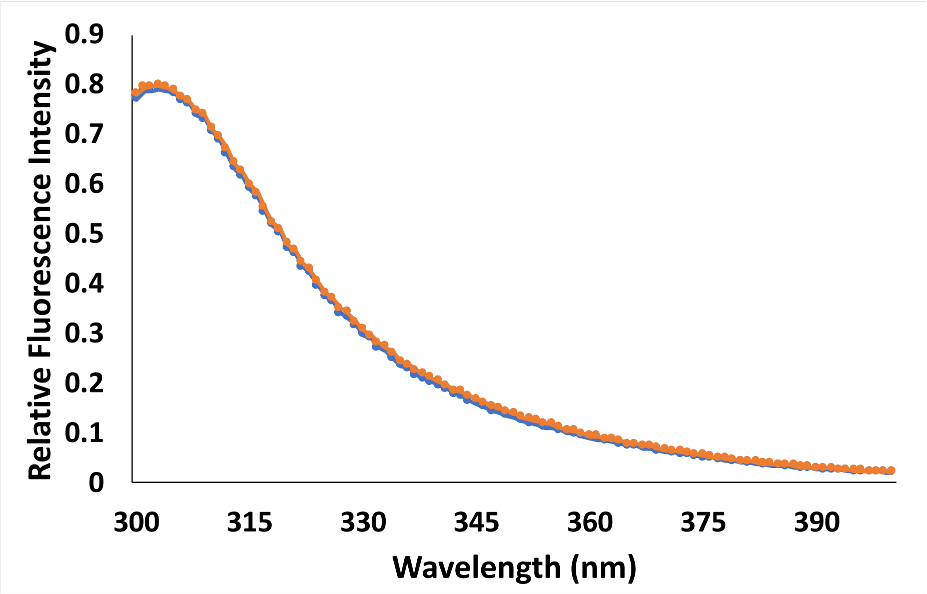

Supplementary Fig. S5.

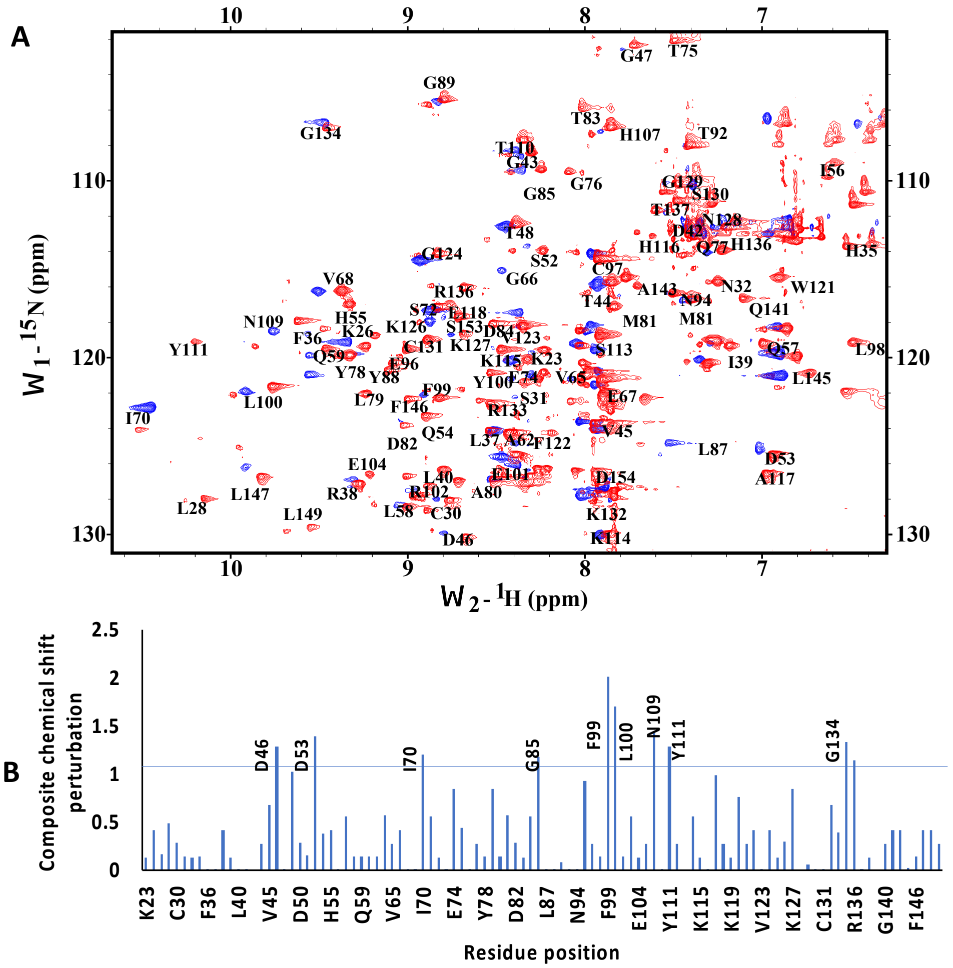

Supplementary Fig. S6.

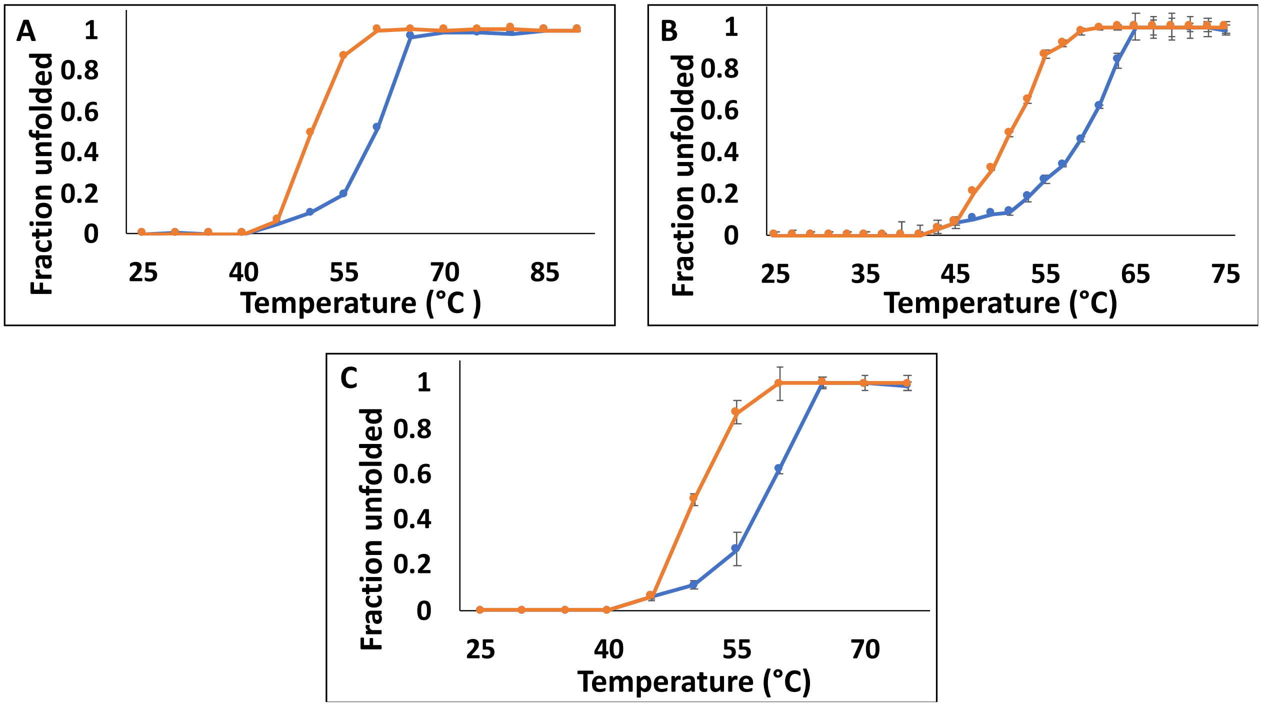

Supplementary Fig. S7.

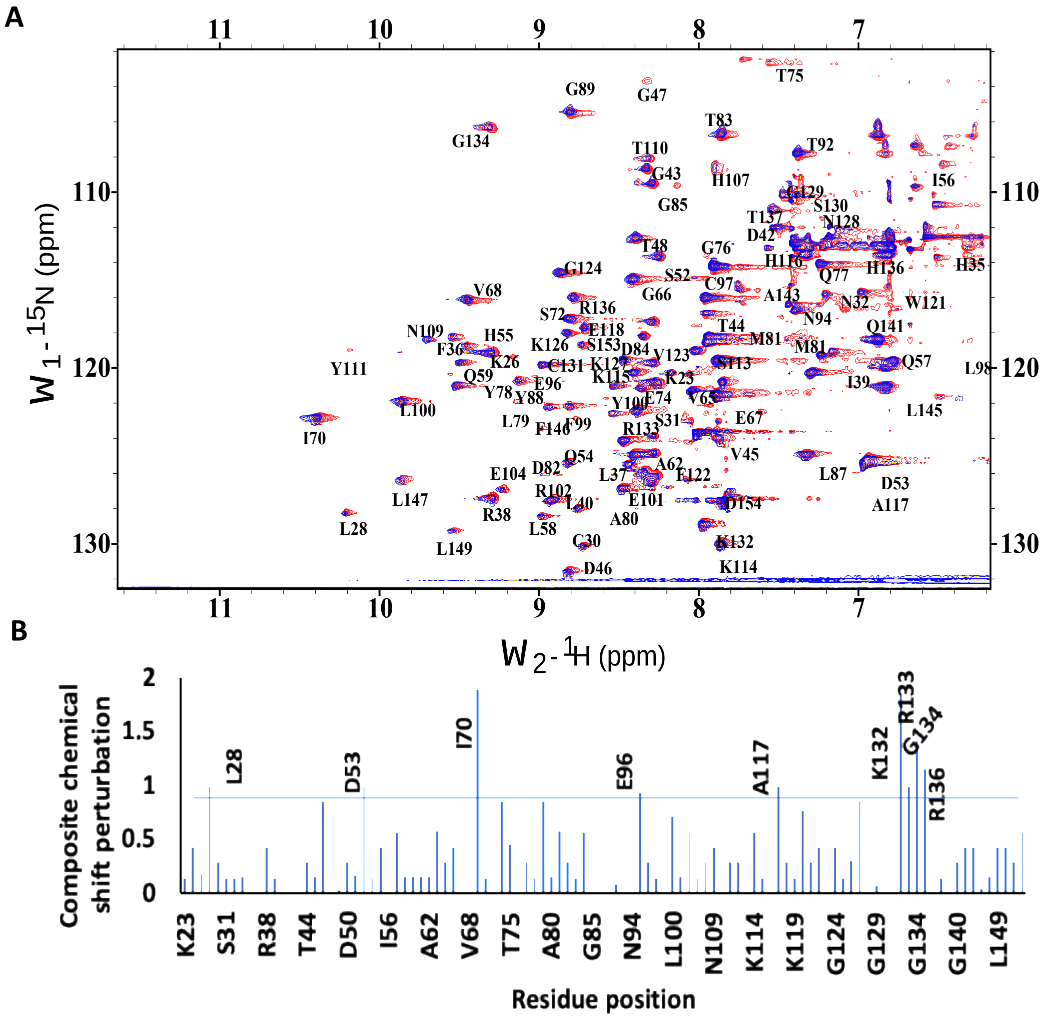

Supplementary Fig. S8.

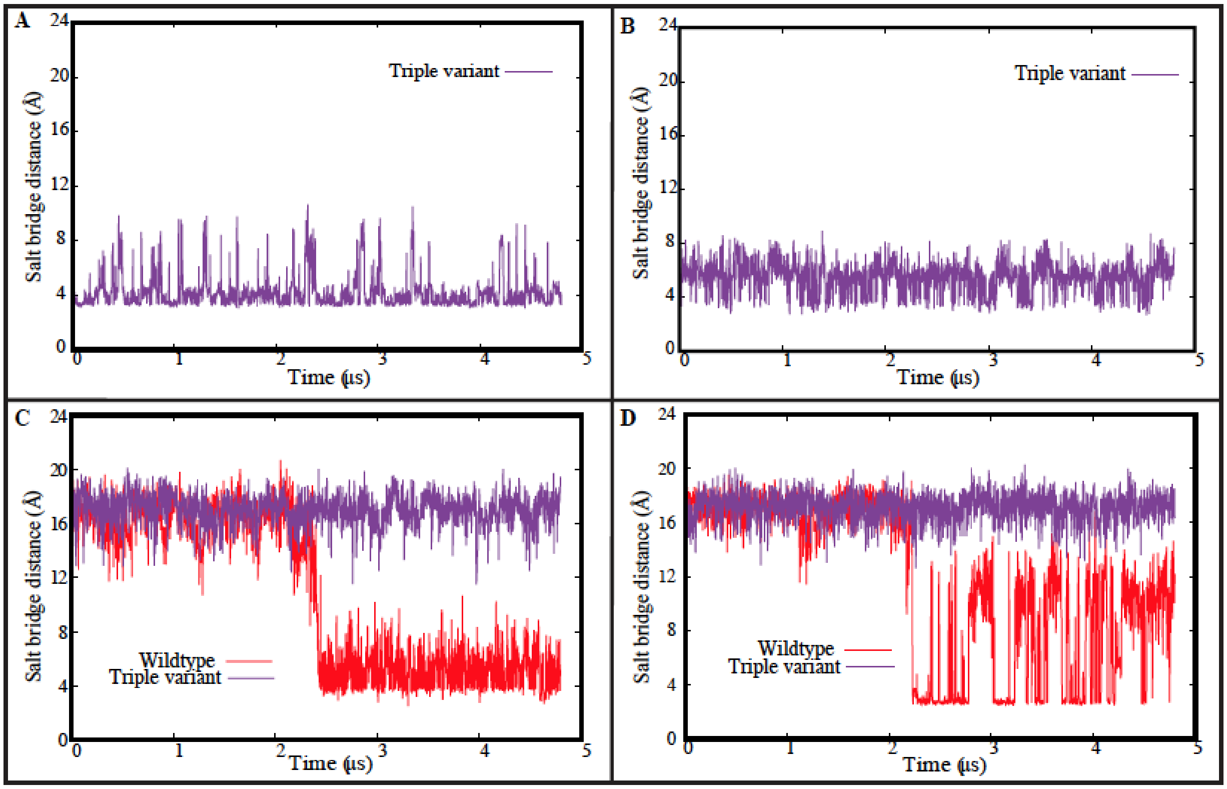

Supplementary Fig. S9.

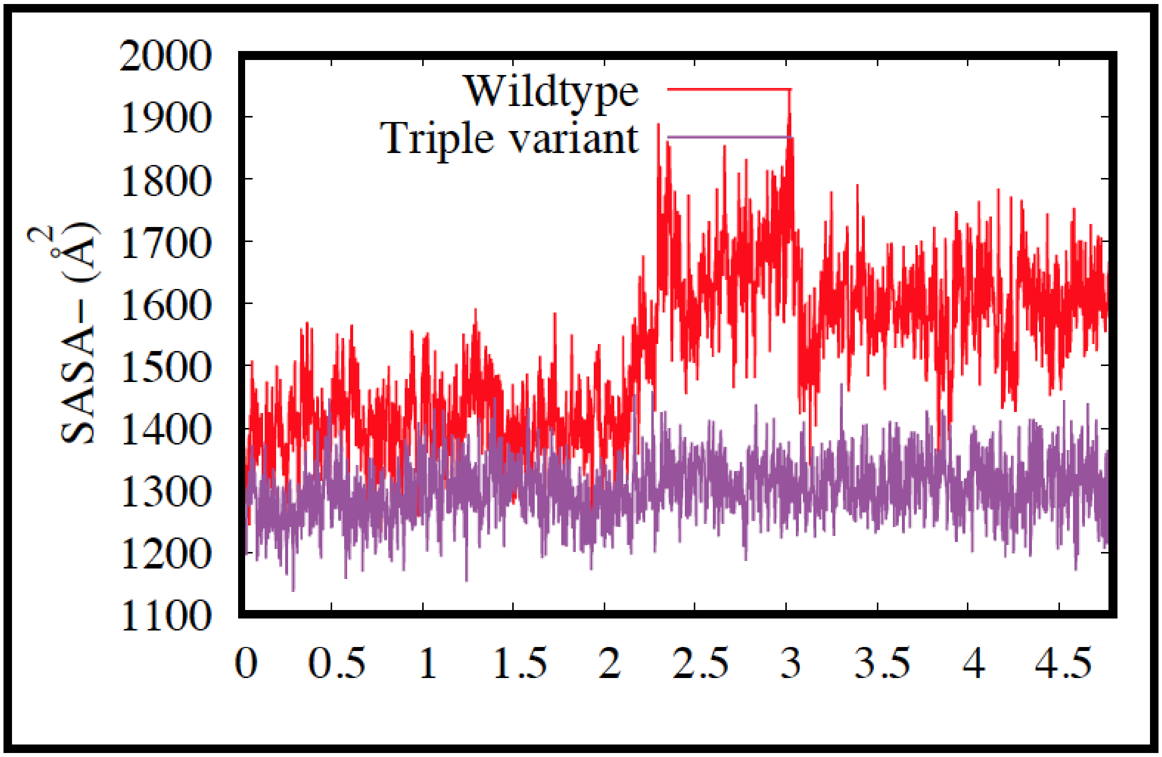

Supplementary Table S1.

Salt bridge/hydrogen bond interaction	Occupancy (%)	
	triple variant	wildtype
**R133 – E136**	**98**	**0**
**N126 – S130**	**79**	**0**
G134 – G85	79	35
T137 – G134	62	35
K132 – G124	62	28
Y139 – E104	54	13
